# Supplementary material for: Splice-Junction-Based Mapping of Alternative Isoforms in the Human Proteome
Source: Cell Rep. Author manuscript; Available in PMC 2020 Jan 15. (PMC6961840; doi:10.1016/j.celrep.2019.11.026)
Supplement: 3 [file NIHMS1546469-supplement-3.zip › DF2/PXD0010154/AdrenalGland-189-Q9BTC8-QVYIPTYKPNPNQISTSNGKPGAVNGAVGTTFQPQNPLLGR.pdf]

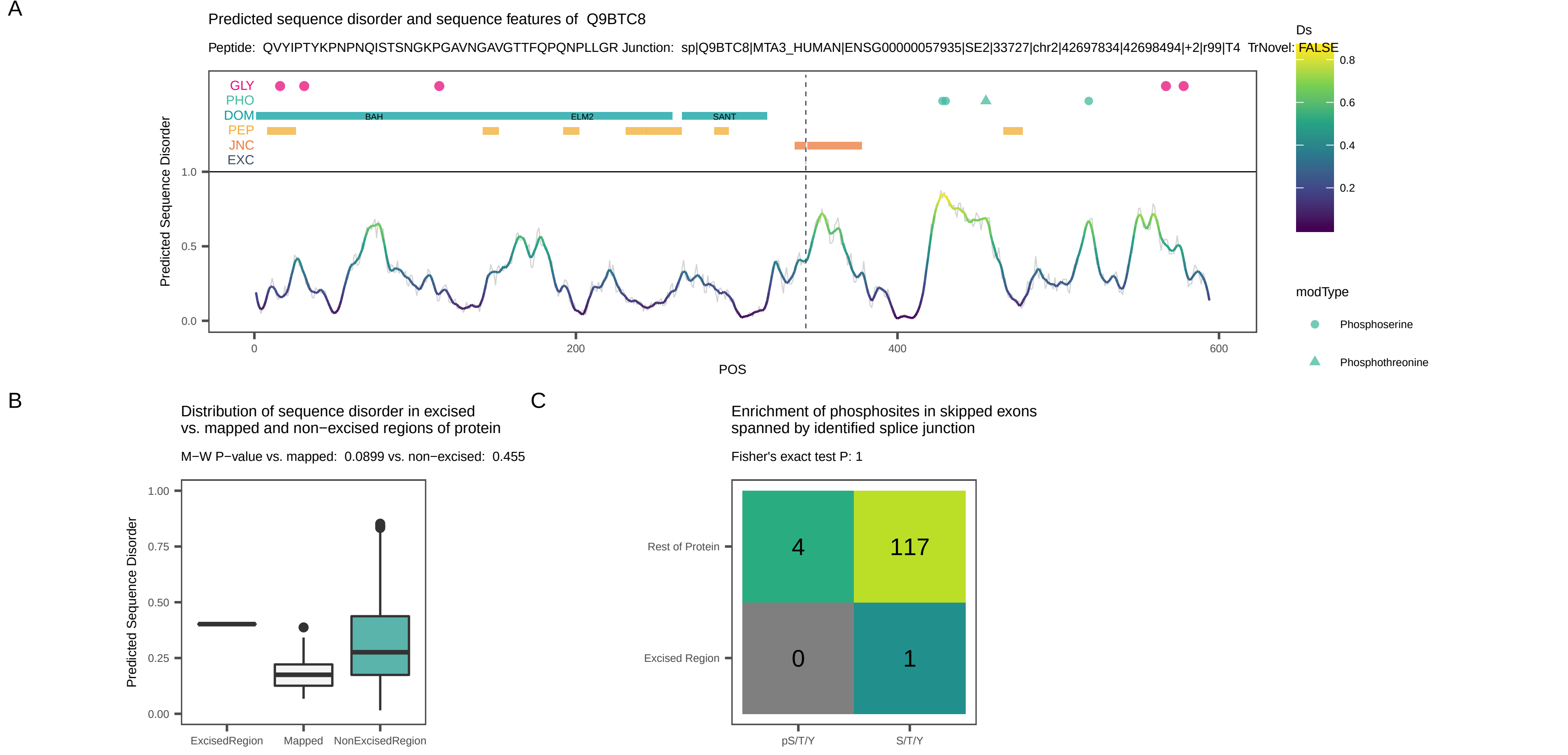
A
Predicted sequence disorder and sequence features of Q9BTC8
Peptide: QVYIPTYKPNPNQISTSNGKPGAVNGAVGTTFQPQNPLLGR Junction: sp|Q9BTC8|MTA3_HUMAN|ENSG00000057935|SE2|33727|chr2|42697834|42698494|+2|r99|T4 TrNovel: FALSE
GLY
PHO
DOM
PEP
JNC
EXC
BAH
ELM2
SANT
Predicted Sequence Disorder
1.0
0.5
0.0
0
200
400
600
POS
Ds
0.8
0.6
0.4
0.2
modType
Phosphoserine
Phosphothreonine
B
Distribution of sequence disorder in excised vs. mapped and non−excised regions of protein
M−W P−value vs. mapped: 0.0899 vs. non−excised: 0.455
Predicted Sequence Disorder
1.00
0.75
0.50
0.25
0.00
ExcisedRegion
Mapped
NonExcisedRegion
C
Enrichment of phosphosites in skipped exons spanned by identified splice junction
Fisher's exact test P: 1
Rest of Protein
Excised Region
4
117
0
1
pS/T/Y
S/T/Y
